# Stimulating proteasomal degradation in human proteinopathies

**DOI:** 10.1111/febs.70638

**Published:** 2026-07-07

**Authors:** Maria E. Gierisch, Enrica Barchi, Nico P. Dantuma

**Affiliations:** ^1^ Department of Cell and Molecular Biology (CMB) Karolinska Institutet Stockholm Sweden

**Keywords:** aggregation, neurodegeneration, proteasome, protein degradation, ubiquitin

## Abstract

The ubiquitin–proteasome system (UPS) comprises hundreds of proteins that orchestrate ubiquitin‐dependent proteasomal degradation and represents a powerful therapeutic target for modulating intracellular protein turnover. Due to its central role in preventing the accumulation of misfolded and dysfunctional proteins, enhancing or suppressing UPS activity offers clinical potential across a wide spectrum of diseases. While oncology has successfully capitalized on this vulnerability through the development of proteasome inhibitors for the treatment of hematological malignancies, efforts to generate clinically relevant UPS activators have progressed more slowly. Bridging this therapeutic gap could be particularly beneficial for neurodegenerative diseases and other proteinopathies, where accelerating the removal of misfolded and aggregation‐prone proteins may help counteract their progressive accumulation and delay, or prevent the onset of symptoms. In this review, we summarize the progress made so far toward finding strategies to boost UPS function through genetic or small‐molecule interventions.

## Graphical abstract

The proteasome mediates the degradation of a wide range of proteins. Boosting proteasomal degradation may be beneficial in several disease contexts and can be achieved either by modulating proteasome activity or by improving substrate delivery. Proteasome activity can be enhanced by increasing proteasome abundance, inducing constitutive gate opening, or engaging alternative regulatory adaptors. Moreover, enhanced substrate delivery can be obtained by facilitating substrate accessibility or through targeted protein degradation strategies.

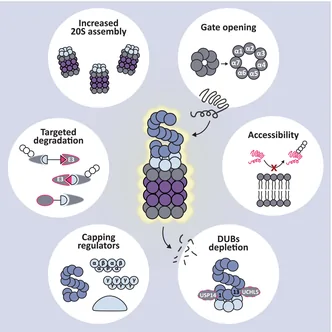

**Abbreviations**
ALS: amyotrophic lateral sclerosis
ARE: antioxidant response element
cAMP: cyclic adenosine monophosphate
cGMP: cyclic guanosine monophosphate
CRBN: cereblon
DUB: deubiquitinating enzyme
ER: endoplasmic reticulum
ERAD: endoplasmic reticulum–associated degradation
FTD: frontotemporal dementia
HD: Huntington's disease
IGF‐1: insulin‐like growth factor 1
MbTAC: Midnolin‐based targeting chimeras
MSP: multisystem proteinopathy
mTOR: mechanistic target of rapamycin
NFE2L1: nuclear factor erythroid 2‐related factor 1
PD: Parkinson's disease
PI3K: phosphoinositide 3‐Kinase
PKA: Protein Kinase A
PKG: Protein Kinase G
PROTAC: proteolysis‐targeting chimera
PSMA: proteasome subunit alpha
PSMB: proteasome subunit beta
PSME: proteasome subunit epsilon
SCA: spinocerebellar ataxia
SDS: sodium dodecyl sulfate
TRX: thioredoxin
UBA: ubiquitin‐associated domain
UBL: ubiquitin‐like protein
UBQLN2: ubiquilin‐2
UPS: ubiquitin‐proteasome system
VCP: valosin‐containing protein
VHL: Von Hippel–Lindau
ZFAND: Zinc‐finger AN1‐type domain

## Introduction

The rapid expansion of aging populations worldwide poses substantial public health challenges, largely driven by the age‐related functional decline and the increased burden of comorbidities such as neurodegenerative diseases [1, 2]. These disorders are rare before midlife, yet their incidence rises sharply beginning in the fifth to sixth decades of life and continues to escalate thereafter [1]. Neurodegenerative diseases share the hallmark feature of progressive neuronal loss, which leads to reduced synaptic connectivity and ultimately impairs brain function. Among them, Alzheimer's disease is the most prevalent worldwide and is defined by the accumulation of β‐amyloid plaques and tau fibrils that progressively disrupt neuronal function [3, 4]. Other relevant examples are Parkinson's disease (PD) [3, 4] and the larger family of polyglutamine diseases, such as Huntington's disease (HD) [3] and spinocerebellar ataxias (SCA) [4], characterized by protein aggregates consisting of α‐synuclein and polyglutamine repeat‐containing proteins, respectively. While neurodegenerative disorders are a prime example, age‐related diseases caused by the accumulation of misfolded proteins are not limited to the nervous system, with other tissues being affected in non‐neurological proteinopathies [5, 6].

A central driver of the progression of age‐related protein aggregation is the gradual loss of proteome integrity, which enables proteins to evade the cellular quality control systems and accumulate in an uncontrolled manner [2, 7]. Over a cell's lifetime, episodes of cellular stress and proteotoxic challenges further exacerbate the buildup of misfolded, damaged, or otherwise dysfunctional proteins [8, 9]. To maintain a healthy proteome, cells must continuously remove these toxic protein species, and their timely destruction represents one of the major challenges for the intracellular degradation systems [10]. Because neurons and other postmitotic cells lack the ability to dispose of or dilute protein aggregates through cell division, they are especially dependent on the long‐term efficiency of protein degradation systems [11]. The persistence of insoluble aggregates, linked to the etiology of specific pathologies, indicates that the combined action of protein quality control and protein degradation systems is ultimately insufficient to clear pathogenic species [7, 11]. This suggests that boosting the UPS's ability to eliminate toxic proteins may hold therapeutic potential.

## Protein quality control by the ubiquitin‐proteasome system (UPS)

**Fig. 1:**
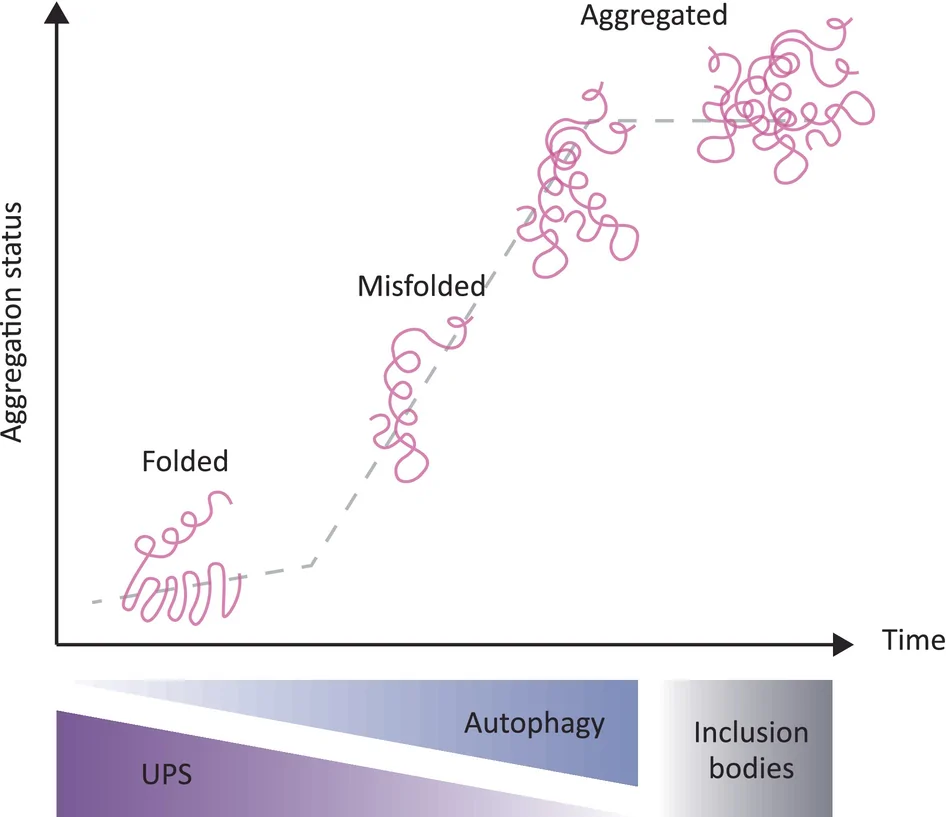
Proteostasis and protein aggregation. The ubiquitin–proteasome system (UPS) handles the turnover of most proteins and can support the degradation of physiological levels of misfolded substrates. When these begin to accumulate and form aggregates, the autophagy machinery takes over their clearance. Finally, if aggregated proteins cannot be processed efficiently, they are sequestered into inclusion bodies and stored for later disposal.

Cells maintain the complexity and integrity of their proteome through a tightly regulated balance between protein synthesis and degradation. When proteins misfold or are no longer needed, they are removed by two dedicated degradation pathways: the UPS or autophagy (Fig. 1). The UPS primarily degrades newly synthesized, short‐lived, or damaged proteins [12], whereas autophagy, in particular macroautophagy, is responsible for clearing insoluble protein aggregates that escape UPS surveillance [13]. If neither system succeeds in eliminating aggregation‐prone proteins and their aggregated forms in a timely manner, these species will gradually accumulate in the intracellular environment. Such buildup will disrupt essential cellular functions and can ultimately lead to cell death. Accordingly, protein aggregates have been shown to possess inherent cytotoxicity [14].

**Fig. 2:**
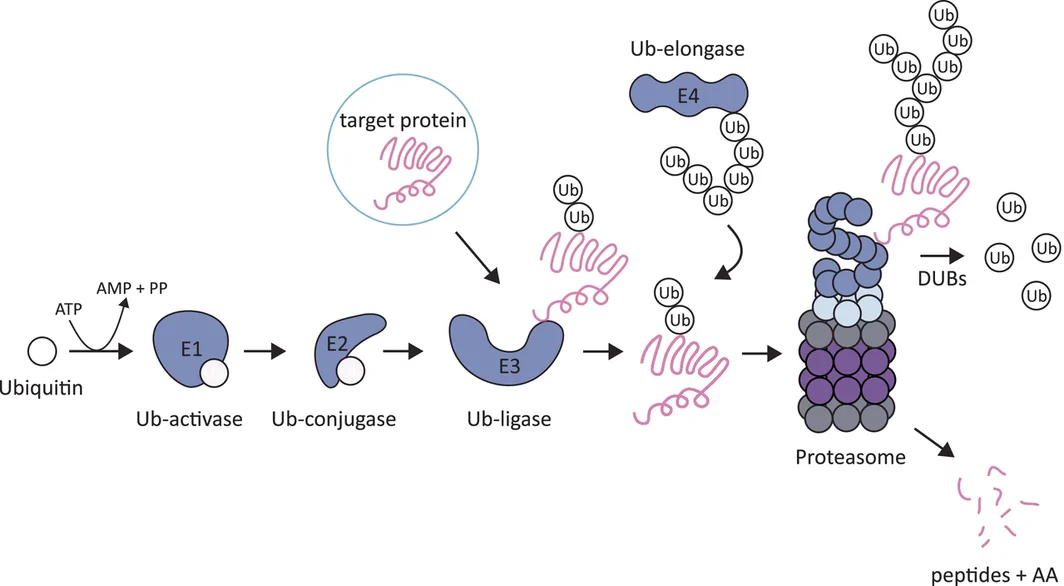
The ubiquitin‐proteasome system. Target proteins are modified with the degradation signal ubiquitin through a coordinated enzymatic cascade, involving ubiquitin‐activases (E1), ubiquitin‐conjugases (E2), ubiquitin ligases (E3), and ubiquitin elongases (E4). Together, these enzymes catalyze the covalent attachment of ubiquitin to the substrate. The repetition of these cycles generates polyubiquitin chains, which serve as signals for recognition and delivery to the proteasome. Proteasome‐associated deubiquitinating enzymes (DUBs) cleave ubiquitin chains, making substrates suitable for entering the proteasome proteolytic channel. Target proteins are then digested into small peptides that can be further recycled by the cells.

The UPS functions as the final safeguard of the cellular protein quality control network, by rapidly eliminating aberrant proteins before they can form these toxic aggregates. Proteins destined for degradation by the UPS are first tagged with the small protein modifier ubiquitin through an enzymatic cascade, involving ubiquitin‐activating enzymes (E1s), ubiquitin‐conjugating enzymes (E2s), and ubiquitin‐ligating enzymes (E3s) [15] (Fig. 2). In some cases, additional ubiquitin chain elongation factors (E4s) can further extend the ubiquitin chain to ensure efficient downstream recognition. During this process, ubiquitin's C‐terminal glycine is, in most cases, conjugated to the ɛ‐amino group of specific lysine residue(s) in the substrate. Moreover, polyubiquitin chains can form when additional ubiquitin molecules are repeatedly conjugated to lysine residues on the ubiquitin already attached to the substrate. This results in the decoration of the protein substrate with either mono‐ or polyubiquitin chains in a process known as ubiquitination. Together with the ubiquitination cascade, a large family of deubiquitinating enzymes (DUBs) provides an additional regulatory layer by trimming, removing or editing ubiquitin chains, thereby modulating the substrate's fate by either delaying or accelerating its degradation [16].

The versatility of ubiquitin arises from the fact that all seven of its lysine residues, and, more rarely, its N‐terminal methionine, can serve as acceptor sites for the carboxy termini of additional ubiquitin molecules. As a result, ubiquitination can generate an extensive variety of linear and branched chains with distinct structural features that are linked to different biological functions. This diversity gives rise to a highly sophisticated ubiquitin *code*, the full complexity of which is only beginning to emerge [17]. While the role of ubiquitination in targeting proteins for proteasomal degradation is the best characterized function of this code, the scope of ubiquitin signaling reaches far beyond the UPS. Among other functions, ubiquitin is also involved in targeting proteins for autophagy, as well as promoting the sequestration of aggregation‐prone proteins in inclusion bodies: two other cellular defense mechanisms against potentially pathological proteins [18].

## The proteasome: A tightly regulated processive protease

**Fig. 3:**
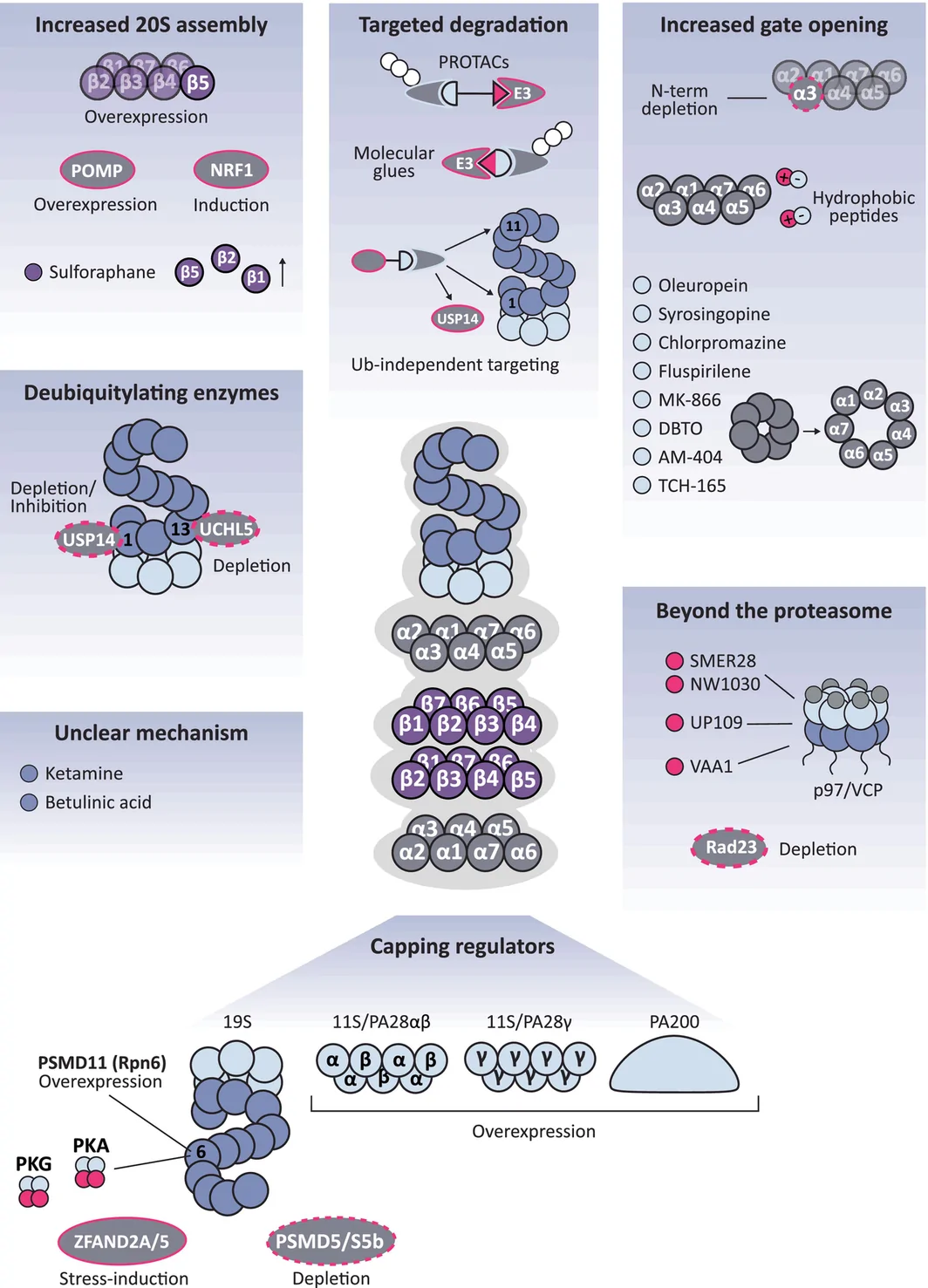
Stimulating proteasomal degradation. Proteasome stimulation can occur through several mechanisms, acting directly or indirectly on the proteolytic machinery, to increase degradation rates of target proteins. The main reported pathways include: (1) enhancing 20S proteasomes assembly by stimulating the synthesis of proteasome subunits; (2) promoting the opening of the α‐ring gate through α3 subunit depletion or via specific peptides and small molecules stimulation; and (3) depleting specific 19S‐associated DUBs. At the capping level, the two 11S complexes and the PA200 monomer serve as alternative lids with proteasome‐activating properties. The 19S regulatory particle can also be modulated by overexpression of Rpn6 or subunits phosphorylation; by induction of the ZFAND protein family members, or by depleting PSMD5/S5b. Beyond the proteasome, p97/VCP stimulation using small molecules enhances proteasomal degradation by promoting unfolding and extraction of target proteins, and Rad23 shuttling‐factor depletion reduces sequestration of ubiquitinated proteins, thereby increasing their accessibility to the proteasome. PROTAC‐ and molecular glue‐based strategies as well as ubiquitin‐independent targeted substrate delivery to the proteasome present strategies that enable a more selective degradation. Finally, ketamine and betulinic acid stimulate proteasome activity through a yet undefined mechanism.

The proteasome is a multi‐subunit proteolytic complex harboring several distinct catalytic activities required for the regulated breakdown of intracellular proteins. Its 20S core particle functions as a compartmentalized protease composed of four stacked heptameric rings that assemble into a cylindrical structure (Fig. 3). The two outer rings consist of seven α‐subunits (PSMA subunits), whereas the two inner rings are formed by seven β‐subunits (PSMB subunits), three of which are proteolytically active. The caspase‐like activity, trypsin‐like activity, and chymotrypsin‐like activity of the PSMB6/β1, PSMB7/β2, and PSMB5/β5 subunits, respectively, are confined to the interior catalytic chamber and endow the proteasome with a broad substrate specificity, enabling it to degrade almost any polypeptide that enters the proteolytic chamber [19, 20]. Stimulation with interferon‐γ induces the expression of three alternative β subunits, PSMB9/iβ1, PSMB10/iβ2, and PSMB8/iβ5, which replace the constitutive catalytic active sites and give rise to immunoproteasomes that retain broad proteolytic capacity [21, 22], but exhibit subtly altered cleavage specificities [23]. The two outer α‐rings regulate the access of substrates to the 20S core with their N termini by obstructing the entrance to the proteolytic chamber [20]: This gating mechanism ensures tight control over proteasomal degradation and prevents unintended proteolysis.

The 20S core particle can associate with several regulatory complexes, each conferring distinct functions and expression patterns. The most abundant is the 19S regulatory particle, a large multi‐subunit assembly responsible for recognizing, unfolding, and translocating ubiquitinated substrates into the proteolytic chamber of the 20S [24]. The 19S regulatory particle consists of two subcomplexes, namely the *base* and the *lid*, which coordinate the substrate processing steps before its degradation [25]. Ubiquitinated substrates are first engaged by the lid, which contains two ubiquitin‐binding subunits: PSMD4/Rpn10 and ADRM1/Rpn13 [26, 27]. Also within the lid, PSMD14/Rpn11 serves as an essential DUB that removes polyubiquitin chains *en bloc*, which is a critical step that allows the substrate to pass through the gated entrance [28]. Two additional DUBs, USP14 and UCHL5, transiently associate with the lid and trim ubiquitin chains from the distal end [29, 30]. The base of the 19S particle consists of two structural proteins, PSMD1/Rpn2 and PSMD2/Rpn1, the former interacting with ubiquitin and ubiquitin‐like (UBL) domains [31, 32], and a ring of six AAA‐ATPases. These ATPases dock onto the entrance of the 20S core, where they facilitate substrate unfolding and translocation into the 20S particle [33]. The base of the 19S also interfaces with the outer α‐rings of the 20S core and induces gate opening through the canonical HbYX motifs (hydrophobic‐tyrosine‐X), present at the C termini of the AAA‐ATPases PSMC1/Rpt2, PSMC4/Rpt3, and PSMC3/Rpt5, which trigger the conformational changes needed to displace the N termini of the α‐subunits that normally occlude the entrance gate [34].

Protein degradation by the 26S proteasome is confined to ubiquitinated substrates and this specificity comes at a high energetic cost, as it requires ATP for protein unfolding and translocation [35]. In contrast, capping of the 20S core with alternative regulatory complexes provides a mechanism for ATP‐ and ubiquitin‐independent proteolysis of substrates that do not require unfolding and can passively diffuse into the proteolytic chamber [36]. Two heptameric 11S regulatory caps can associate with the 20S core in specific physiological conditions and form ring‐like structures that modulate substrate access [37, 38, 39]. Binding of either the interferon‐inducible cytosolic PSME1‐2/PA28α‐β 11S or the constitutive nuclear PSME3/PA28γ 11S complexes induces gate opening and shifts substrate preference toward intrinsically disordered, oxidized or unfolded proteins [37, 40, 41]. In addition to these ring‐shaped regulators, the predominantly nuclear 200 kDa PSME4/PA200 complex serves as another 20S regulatory particle [42]. Due to its high expression levels in the testis, it has been proposed to play a major role in spermatogenesis [42, 43]. The PSME4/PA200‐capped 20S only partially opens the gate, resulting in a complex that has a strong preference for acetylated histones and similarly sized unfolded proteins [44].

Despite the diversity of regulatory complexes that can associate with the 20S core, the 19S regulatory particle remains the only proteasome adaptor capable of facilitating ubiquitin‐dependent degradation. Its ability to recognize ubiquitinated proteins depends on both the linkage type and the architectural complexity of the ubiquitin chain [45]. Most ubiquitin linkages can target proteins for proteasomal degradation, with the notable exception of homotypic K63‐linked chains [46], which generally participate in autophagy and nonproteolytic processes. Among the degradation‐competent ubiquitin signals, K48‐linked and branched ubiquitin chains are the principal marks detected by the proteasome [47]. Homotypic K48‐linked chains are recognized predominantly by the PSMD4/Rpn10 subunit, whereas branched chains with diverse topologies engage the proteasome through the simultaneous binding of multiple ubiquitin receptors [48]. Ubiquitin receptor occupancy allosterically modulates the proteasomes, driving it into engagement‐ and processing‐competent states that promote efficient substrate unfolding and translocation by the 19S AAA‐ATPases [49]. Once the substrate is properly engaged, the DUB PSMD14/Rpn11 removes the ubiquitin chain, thereby committing the substrate to proteasomal degradation [50, 51].

Several auxiliary proteins assist the proteasome by preprocessing, extracting, or delivering ubiquitinated substrates. Shuttling factors such as hRad23A/B and the ubiquilins selectively recognize ubiquitinated proteins and deliver them to the proteasome for degradation [52]. Equipped with both a UBL domain and ubiquitin‐associated (UBA) domain, these proteins can simultaneously capture ubiquitinated substrates and bind to the proteasome, thereby ensuring efficient delivery of a broad range of substrates. Another assisting factor is the ubiquitin‐selective unfoldase p97/Valosin‐containing protein (VCP), which is an AAA‐ATPase that facilitates proteasomal degradation by extracting ubiquitinated substrates from membranes or multiprotein complexes, or by initiating the unfolding of substrates to promote efficient handover to the proteasome [53].

During proteotoxic stress, several proteins support the 19S regulatory particle in handling the type of substrates that typically accumulate in these conditions. One such factor is the redox‐active thioredoxin (TRX)‐like protein TXNL1, which binds the PSMD14/Rpn11 subunit of the 19S particle and is thought to facilitate proteasomal degradation, likely by reducing oxidized substrates during the unfolding process [54]. Another transient interactor is Zinc Finger AN1‐type Domain family 5 (ZFAND5), which increases the residence time of ubiquitinated substrates at the 19S and enhances engagement by the AAA‐ATPases [55, 56]. Upon ZFAND5 dissociation, the proteasome undergoes conformational changes at its entrance channel that promote efficient translocation of the captured substrate [55, 56]. Beyond factors that influence substrate handling, additional proteins modulate the structural composition of the 26S proteasome. Notably, ECM29 promotes its disassembly, separating the 19S regulatory particle from the 20S core [57]. This separation changes the dynamics of proteasomal degradation by shifting them toward the ubiquitin/ATP‐independent degradation by the 20S core particle [57, 58].

## Boosting proteasome levels

Among the three catalytically active proteasome subunits, the chymotrypsin‐like activity of PSMB5/β5 is the dominant driver of protein degradation under steady‐state conditions [59]. Since its inhibition strongly suppresses overall proteasomal degradation rates, several studies have explored the effect of overexpressing PSMB5/β5 (Fig. 3). For this subunit to be proteolytically active, it must be incorporated into a fully assembled 20S proteasome, together with active PSMB6/β1 and PSMB7/β2 subunits. Overexpression of PSMB5/β5 in multiple cellular models consistently leads to increased levels of chymotrypsin‐like processing, accompanied by an overall increase in assembled proteasomes [60, 61, 62, 63, 64]. As expected based on the stoichiometry of the active subunits in the mature proteasomes, the other two catalytic activities are also enhanced upon ectopic expression of PSMB5/β5 [60, 61, 62, 64]. These effects correlate with accelerated degradation of polyubiquitinated substrates and greater resistance to stressors, such as oxidative stress [60, 62]. Additional studies show that PSMB5/β5 overexpression can counteract the age‐related decline in proteasome function and restore overall proteasome capacity in patient‐derived aged dermal fibroblasts [61], human mesenchymal stem cells [64], and senescent bone‐marrow stromal cells [63]. Subsequently, this reduces the accumulation of oxidized and ubiquitinated proteins while improving resistance to stress conditions [61, 63].

Extending these findings to whole‐organism models, PSMB5/β5 overexpression in *Caenorhabditis elegans* [65] and *Drosophila melanogaster* [66, 67] likewise leads to enhanced chymotrypsin‐like proteasomal activity. Consistently with results in cells, these *in vivo* aging models exhibit increased levels of proteasome subunits and improved overall proteolytic capacity, which correlate with extended lifespan and greater resistance to oxidative stress [65, 66]. Both in nematodes and fruit flies, overexpression of the PSMB5/β5 subunit resulted in an increase in assembled proteasomes [65, 67], despite it having a limited effect on the transcription of the other core subunits [65, 66, 67].

Beyond single‐subunit overexpression, activation of endogenous regulatory pathways that drive proteasome assembly provides another strategy to enhance proteasome capacity as for overexpression of POMP, a dedicated assembly chaperone that is required for the formation of the 20S core particle [68]. In human fibroblasts, POMP overexpression increases proteasomal subunit protein levels and proteasome activity [69]. Moreover, in the context of oxidative stress, cells overexpressing POMP exhibit increased viability and a more rapid restoration of proteasome function during the recovery phase [69, 70].

Nuclear Factor Erythroid 2‐related factor 1 (NFE2L1, also known as NRF1) is a transcription factor that upregulates the expression of genes encoding proteasome subunits in response to impaired proteasome activity, typically triggered by proteotoxic stress [71]. Being the master regulator in a coordinated program to induce the expression of most proteasome subunits, NRF1 binds antioxidant response elements (ARE) at promoters in the nucleus [71, 72]. There is currently no evidence of a selective preference for specific subunits, such as the catalytic β‐subunits. In fact, NRF1 induction leads to transcriptional upregulation of both 20S and 19S subunits, as well as the alternative PSME4/PA200 cap, the proteasome assembly factor POMP, and the segregase p97/VCP [73], indicating that the inherent subunits' stoichiometric ratios must be preserved to increase proteasome abundance. This adaptive feedback mechanism helps to restore protein homeostasis and can be experimentally leveraged to stimulate proteasomal function. While NRF1 suppression has been explored as a way to boost the effectiveness of proteasome inhibitors in multiple myeloma treatment [74], enhancing NRF1 activity in the context of neurodegenerative diseases represents a promising therapeutic approach to reduce the accumulation of aggregation‐prone or oxidized proteins. For example, the synthetic curcumin analog ASC‐JM17 upregulates NRF1 expression, thereby increasing overall proteasome abundance and lowering the levels of the aggregation‐prone mutant androgen receptor protein that causes the polyglutamine disorder spinal and bulbar muscular atrophy [75]. RUN‐47, which was identified through a high‐throughput chemical screen as an NRF1 transcriptional activator, stimulates proteasome activity through a mechanism similar to that of ASC‐JM17 and has been shown to reduce the levels of aggregation‐prone α‐synuclein and mutant huntingtin [76].

## Opening the gate

As previously mentioned, the outer α‐rings form the 20S substrate translocation channel, with their N‐terminal tails occluding the central pore and thereby regulating access to the 20S core particle. Substrate engagement by the 19S regulatory particle induces displacement of these N termini, resulting in the opening of the entrance gate [77]. Early studies using the detergent sodium dodecyl sulfate (SDS) suggested that proteasomal gate‐opening can be induced by disrupting the structural interactions that normally maintain the α‐ring in a closed conformation [78]. Although SDS has been proven useful as a tool *in vitro* and in some cell culture settings, it is not suitable as a therapeutic agent. Nonetheless, these findings motivated additional efforts to identify alternative strategies to achieve similar gate‐opening effects under physiologically relevant conditions.

Constitutively open‐gate proteasomes have been engineered by genetically deleting the N terminus of the PSMA4/α3 subunit, a structural element crucial for shaping and stabilizing the closed conformation of the 20S gate (Fig. 3). Removal of this N‐terminal part prevents the conformational changes required for gate closure, leaving the α‐ring permanently open [20, 79, 80, 81]. In cells, these open‐gate proteasomes exhibit increased hydrolysis of small fluorogenic substrates [79], which are commonly used to assess proteasome activity *in vitro* [82]: the enhanced cleavage of these small peptides reflects, in this context, increased accessibility of the proteolytic sites, rather than an intrinsic change in their catalytic activity. Because these small fluorogenic substrates do not require unfolding or translocation, the elevated activity of open‐gate proteasomes may preferentially promote the degradation of proteins that are less reliant on unfolding.

Nevertheless, it has been shown that some ubiquitinated substrates are also processed faster by proteasomes with constitutively open gates [79]. For instance, deletion of the highly conserved PSMA4/α3 N terminus in *C. elegans* also resulted in increased proteolytic processing and enhanced clearance of misfolded and aggregation‐prone proteins [80, 81]. In this context, open‐gate proteasomes contributed to the enhanced degradation of endoplasmic reticulum‐associated degradation (ERAD) substrates, a class of proteins that undergoes partial unfolding in the process of p97/VCP‐dependent extraction from the endoplasmic reticulum (ER) [81], confirming that, when extraction and unfolding are not rate‐limiting, the accelerated degradation supported by the open‐gate proteasome extends to physiological substrates. The specificity of open‐gate proteasomes therefore remains uncertain, and such interventions may ultimately alter the overall *degradome* (i.e., the total pool of degraded proteins). Proteasome hyperactivation via open‐gate strategies in *C. elegans* extends lifespan and enhances resistance to oxidative and proteotoxic stresses, but this benefit comes at the cost of markedly reduced reproductive output [80, 81].

Early studies using hydrophobic peptide substrates initially suggested that these molecules could allosterically activate the proteasomes' proteolytic sites [83], but subsequent observations were inconsistent with this model [84]. Instead, later work demonstrated that the enhanced activity observed with these peptides resulted from their ability to promote opening of the 20S proteasome gate [85]. Building on the idea that persistent 20S gate opening can be achieved by modulating structural elements within the α‐ring gating complex, a rational approach was adopted. This method focuses on generating peptides that bind to the intersubunit pockets of the α‐rings and displace the N‐terminal gating residues, thereby triggering structural changes and stabilizing a constitutively open‐gate conformation. The endogenous AAA‐ATPases subunits of the 19S regulatory particle use precisely this mechanism, thanks to the HbYX motifs in their C‐terminal tails [77, 86]. Inspired by this natural mechanism, synthetic HbYX‐like peptides have been developed that can occupy the α‐ring pockets and induce persistent gate opening, even in the absence of the 19S regulatory particle.

Using the minimal structural features required for HbYX‐mediated binding, a dipeptide was identified that is sufficient to induce gate opening, stimulate proteasomal processing and promote tau degradation [87]. Notably, this peptide also rescued impaired proteostasis caused by exogenously administered mutant huntingtin or Aβ‐42 oligomers [87]. In addition to artificial peptides, natural HbYX motifs, such as those of PSMC3/Rpt5, have also served as templates for engineering gate opening molecules that enhance the degradation of α‐synuclein and tau *in vitro* [88]. Due to the generally limited cell permeability of peptides, the PSMC3/Rpt5 analog was fused to a cell‐penetrating peptide, TAT, to promote the crossing of the plasma membrane, resulting in enhanced proteasomal processing in cells [88]. Other peptides capable of enhancing proteasomal activity in a similar manner include those engineered from other natural 20S regulators, such as the C‐terminal fragment of the PSME4/PA200 cap [89]. Alternative strategies include the conversion of allosteric inhibitors of the 20S core [90] to activators by appending the HbYX motif [91] and selecting peptides with specific features, such as cyclic peptides, that increase their stability and cell permeability [92]. Other peptides have been further optimized through rational design following their initial identification, such as PAP1, which enhances proteasomal activity and reduces the levels of both oxidized proteins and the insoluble fraction of SOD1 [93].

Beyond peptides, several small‐molecule compounds have been found to stimulate proteasome activity. Oleuropein was among the first compounds reported to enhance 20S proteasomal activity *in vitro* and induce a delayed senescence phenotype in human embryonic fibroblasts under continuous treatment [94]. Following this line of research, high‐throughput screens have identified several small molecules that induce an open‐gate conformation of the 20S proteasome, including Chlorpromazine [95], TCH‐165 [96], Fluspirilene and its derivatives [97], and Syrosingopine [98]. For Chlorpromazine, TCH‐165, and Fluspirilene, docking studies have mapped their binding sites to the pockets located between the PSMA6/α1 and PSMA2/α2 [95, 96] subunits, or between the PSMA2/α2 and PSMA4/α3 subunits [97]. *In vitro*, all the compounds increased the proteasomal enzymatic activities and enhanced the degradation of the recombinant and aggregation‐prone proteins α‐synuclein [95, 96, 97, 98] and tau [95]. Other screenings identified 20S activators such as AM‐404, MK‐866 [99] and DBTO ((1R,3E,6R,7Z,11S,12S)‐dolabella‐3,7,18‐trien‐6,17‐olide) [100], by using fluorogenic peptide assays to detect increased opening of the 20S proteasome gate and further validated their effects on aggregation‐prone proteins, such as α‐synuclein [99] and Aβ‐42 [100] in human cell lines.

## Assembling the 19S regulator

Under physiological conditions, proteasomes exist primarily as 20S core particles with 19S regulatory complexes docked to one or both of their entry channels, forming 26S or 30S proteasomes respectively. The six PSMC/Rpt ATPase subunits that make up the base of the 19S regulatory particle hydrolyze ATP to drive unfolding and translocation of the substrate into the 20S proteolytic chamber [24]. Mechanistically, these ATPases may be suitable targets for enhancing the degradation of aggregation‐prone proteins. This concept stems from the observation that their unfoldase activity is less efficient when processing substrates enriched in low‐complexity amino acid sequences [101, 102]. Potentiating the ability of the PSMC/Rpt ATPases in handling ‘slippery’ substrates could, in principle, improve the clearance of aggregation‐prone proteins that can otherwise cause cellular stress by interfering with the translocation machinery. However, the coordinated and highly dynamic nature of substrate recognition, mechanical unfolding and vectorial translocation into the 20S chamber presents substantial challenges for therapeutic interventions targeting this pathway.

On the other hand, several studies have demonstrated that altering the expression levels of the 19S non‐ATPase subunits can enhance proteasomal degradation [103, 104, 105, 106]. One such subunit, PSMD11/Rpn6, plays a crucial role in stabilizing the interaction between the 19S regulator and the 20S core particle [107]. In *C. elegans*, germline‐deficient strains exhibit extended lifespan and elevated levels of chymotrypsin‐like activity, a phenotype attributed to increased levels of the PSMD11/Rpn6 orthologue [103]. Although overexpression of PSMD11/Rpn6 in otherwise normal worms does not reproduce the longevity phenotype, it improves stress resistance and enhances the clearance of protein aggregates. In mammalian systems, PSMD11/Rpn6 expression levels also similarly correlate with proteasomal capacity [103]. During differentiation of human embryonic stem cells into mature neurons, the decline in PSMD11/Rpn6 protein levels correlates with reduced abundance of assembled 19S regulatory complexes and diminished proteasome activity. Neuron‐specific overexpression of PSMD11/Rpn6 restores these deficits, increasing the levels of both single‐ and double‐capped proteasomes and boosting overall proteasomal activity [104].

Not only overexpression, but also depletion of 19S interactor proteins can stimulate proteasomal activity. PSMD5/S5b functions as a 19S assembly chaperone that transiently stabilizes the pre‐assembled complex but dissociates once the mature 26S proteasome has been formed [108]. PSMD5/S5b binds several 19S subunits, including PSMC1/Rpt2, PSMC2/Rpt1, and PSMD2/Rpn1, and suppresses the ATPase activity by stabilizing these intermediates, thereby delaying 26S proteasome assembly [105, 108, 109]. Depletion of PSMD5/S5b enhances proteasomal degradation, increases lifespan in *Drosophila*, and reduces the levels of aggregated tau in a tauopathy model [105]. Similarly, mice lacking PSMD5/S5b show elevated formation of both single‐ and double‐capped 26S proteasomes and display enhanced clearance of diverse aggregation‐prone proteins, such as SOD1, TDP‐43, FUS, and tau [106].

A variety of nonproteasomal proteins transiently associate with the 19S regulatory particle, and several of these factors modulate substrate selectivity or influence overall proteasome activity. Prominent among them are members of the ZFAND family, a group of stress‐responsive proteins that are recruited to the 26S proteasome [110]. Although their basal expression is typically low or restricted to specific tissues, their levels rise sharply in response to stress signals. For example, ZFAND2A/AIRAP is strongly induced by arsenite‐triggered oxidative stress [111], whereas the previously mentioned ZFAND5 is upregulated during fasting [112]. In both cases, this induction has been shown to enhance proteasomal activity and protein degradation [56, 111, 112]. Mechanistically, ZFAND2A/AIRAP binds to the 19S regulatory particle near the PSMD2/Rpn1 subunit, a positioning that promotes increased proteolytic output of the 26S proteasome. Consistent with this activating function, *C. elegans* or mouse embryonic fibroblasts lacking ZFAND2A/AIRAP exhibit reduced 26S proteasome activity accompanied by the accumulation of ubiquitinated substrates [111].

Although multiple ZFAND proteins share the ability to stimulate proteasome activity under stress conditions, it remains unclear to what extent they operate through similar molecular mechanisms. The exact mechanism by which ZFAND2A/AIRAP enhances proteasome function is still poorly defined, but a more detailed understanding has emerged for ZFAND5 [55, 56]. Upon stress induction, ZFAND5 binds to the 19S subunits PSMC3/Rpt5, PSMC2/Rpt1, and PSMD2/Rpn1, leading to a widening of the substrate entry channel into the 20S core. By simultaneously recruiting ubiquitinated substrates and prolonging their dwell time at the proteasome, ZFAND5 increases the likelihood that substrate engagement results in productive degradation, an effect particularly important for substrates that are otherwise difficult to process [55]. In cell lysates, the addition of ZFAND5 leads to an increase in catalytic activities of the proteasome, together with an overall enhanced protein degradation rate [56]. Several additional ZFAND family members have been reported to associate with either the proteasome [110] or p97/VCP [110, 113], suggesting that they may act as important adaptor proteins, but their mechanisms of action and regulatory roles remain largely unresolved.

## Posttranslational modifications of the proteasome

Proteasome subunits undergo posttranslational modifications, such as O‐GlcNAcylation [114], ADP‐ribosylation [115, 116], and phosphorylation [117, 118, 119]. These modifications occur both under steady‐state conditions and in response to physiological cues. Phosphorylation, in particular, affects subunits of both the 20S core and the 19S regulatory particle. As phosphorylation is reversible and mediated by defined kinases, it represents an attractive regulatory node for therapeutic intervention.

Among the known signaling pathways, elevated intracellular cyclic adenosine monophosphate (cAMP) levels and activation of protein kinase A (PKA) have been most consistently linked to proteasome activation. PKA‐dependent phosphorylation of the proteasome, predominantly targeting serine 14 of PSMD11/Rpn6 [120], has been shown to stimulate all three catalytic proteasomal activities, both *in vitro* and in cellular systems [120, 121, 122, 123, 124]. The cAMP–PKA pathway can be activated through treatment with dibutyryl‐cAMP or pharmacological activators, such as Forskolin or Rolipram, which leads to increased abundance of double‐capped proteasomes [120] and accelerated degradation of UPS reporter substrates and aggregation‐prone proteins, such as tau and α‐synuclein [120, 122]. In a tauopathy mouse model, in particular, elevating cAMP‐PKA signaling attenuated compromised proteasome function and reduced tau accumulation, demonstrating the therapeutic potential of modulating this pathway [123]. Physiologically, endogenous cAMP levels can rise in several contexts, including during exercise in muscle cells, short‐term fasting in the liver and hormonal stimulation of hepatocytes by epinephrine or glucagon [125]. In each of these settings, elevated cAMP activates PKA, leading to phosphorylation of PSMD11/Rpn6 and a corresponding increase in proteasomal activity [125].

In addition to the cAMP‐PKA axis, the activation of cyclic guanidine monophosphate of protein kinase G (cGMP‐PKG) signaling pathway also enhances proteasomal activity through the phosphorylation of a yet‐unidentified 26S proteasome subunit [126]. This activation has been linked to increased degradation of both short‐ and long‐lived proteins, an effect partially attributed to increased substrate ubiquitination [126]. Pharmacologically elevated cGMP–PKG signaling, induced by compounds such as Tadalafil or Sildenafil, reduces the levels of mutant CryAB in desmin‐related cardiomyopathy [127], as well as tau and mutant huntingtin levels in neurodegenerative diseases [126, 127]. Thus, activation of either the cAMP/PKA or cGMP/PKG pathways stimulates proteasomal activity and promotes the clearance of aggregation‐prone proteins.

In an unbiased high‐throughput cell‐based screen for stimulators of proteasomal degradation, inhibitors of the stress‐activated kinase p38 MAPK were found to enhance ubiquitin‐dependent degradation [128]. This effect is consistent with previous studies, showing that inhibition or depletion of proteins acting upstream or downstream of p38 MAPK leads to a similar stimulatory effect. Moreover, it has been observed that osmotic stress exposure in cells results in p38 MAPK‐dependent phosphorylation of proteasome subunits and reduced proteolytic activity [129]. However, the enhanced degradation rates from p38 MAPK inhibition are not due to changes in phosphorylation status or increased expression of proteasome subunits [128]. The exact molecular mechanism by which proteasome efficiency is improved therefore remains to be elucidated. Nevertheless, the fact that inhibition of p38 MAPK preferentially accelerates the degradation of short‐lived and aggregation‐prone proteins, combined with its role in stress responses and the targetable nature of this pathway suggests that it may be an interesting strategy warranting further exploration [130].

Upon treatment with the hormone insulin‐like growth factor‐I (IGF‐I), proteasomal activity is enhanced both in cells and *in vivo* without any detectable change in the abundance of proteasome subunits, leading to increased degradation of oxidized and damaged proteins [131]. This response has been proposed to result from proteasome phosphorylation mediated by phosphoinositide 3‐kinase (PI3K) and mechanistic target of rapamycin (mTOR), since pharmacological inhibition of either kinase abolishes the effect [131]. In contrast, under steady‐state conditions, mTOR inhibition elicits a different response and rapidly stimulates ubiquitin‐dependent proteasomal degradation [132].

## Targeting the DUBs of the 19S regulator

The three deubiquitinating enzymes PSMD14/Rpn11, USP14, and UCHL5 are positioned at distinct sites on the 19S regulatory particle and control the processing of the substrate prior to degradation [133]. While PSMD14/Rpn11 is an integrated subunit, USP14 and UCHL5 only transiently associate with the regulatory particle. Upon binding of a ubiquitinated substrate to USP14's UBL domain, a conformational shift is triggered that promotes substrate entry into the 20S core channel [134]. Overexpression of the UBL domain of USP14 is sufficient to enhance proteasomal degradation, a mechanism that also holds true for other proteins with similar domains [135].

The small‐molecule USP14 inhibitor IU1 mimics the phenotype of USP14‐deficient cells by stimulating all three proteasomal catalytic activities and promoting the clearance of aggregation‐prone, oxidatively damaged proteins and aberrant nascent proteins [136, 137]. Moreover, IU1 has also been shown to prevent cell death in an *in vitro* ischemia model [138]. The more potent derivative IU1‐47 stimulates degradation of both wild‐type and mutant tau in primary neuronal cultures [139]. Notably, IU1‐47 also boosts the autophagic flux [139] and stimulates parkin‐independent mitophagy [140], indicating effects beyond proteasomal activation.

The proteasome‐associated deubiquitinating enzyme UCHL5 binds to the ubiquitin‐receptor subunit PSMD16/Rpn13. UCHL5, similarly to USP14, trims ubiquitin chains from the distal end, with a strong preference for K48‐linked branched ubiquitin chains [19, 141]. In a long‐lived *C. elegans* strain with reduced insulin/IGF‐1 signaling, elevated catalytic proteasome activity was observed to correlate with decreased protein levels of the nematode orthologue of UCHL5 [142]. In human cells, depletion of UCHL5 levels also resulted in reduced levels of an engineered UPS reporter substrate, as well as ectopically expressed wild‐type and mutant ataxin‐3 [142].

PSMD14/Rpn11 is a constitutive and essential subunit of the 19S regulatory particle and acts on substrates that are committed to degradation by cleaving ubiquitin chains *en bloc* [30]. The binding of the substrate's ubiquitin chain to PSMD14/Rpn11 results in the stabilization of an engagement‐competent state of the proteasome [50, 51]. As such, PSMD14/Rpn11 is critical for efficient proteasomal degradation, because any remaining ubiquitin molecules on the substrate will hinder its translocation through the narrow entrance pore. Indeed, depletion or inhibition of PSMD14/Rpn11 was found to delay the degradation of substrates *in vitro* [28, 30]. While PSMD14/Rpn11 depletion is generally toxic, its overexpression in a *Drosophila* model rescued the age‐related reduction in catalytic proteasomal activities and extended the lifespan [143]. Given the stoichiometry of a single PSMD14/Rpn11 molecule per 19S regulatory particle [24] and the fact that Rpn11 is only fully catalytically active when incorporated into the 19S [144], the enhanced activity is likely due to an increased abundance of 19S‐capped proteasomes.

## Mobilizing other 20S cap regulators

The 20S core can also associate with alternative regulators that lack ubiquitin binding sites, DUBs, and AAA‐ATPases of the 19S particle. These alternative caps include the 11S/PA28 family (PSME1–3) complexes and the PSME4/PA200 proteasome activator, which play a more passive but still important role in proteasomal degradation [38, 145, 146].

PSME1/PA28α and PSME2/PA28β form a hetero‐heptameric ring that docks onto the 20S entrance [36, 41]. Although the PA28αβ complex was originally thought to function predominantly in antigen presentation by promoting the generation of peptides suited for loading onto MHC class I molecules [37, 147, 148], it is now clear that it is also involved in the enhanced degradation of proteins that accumulate during proteotoxic insults [146]. For instance, in a desmin‐related cardiomyopathy model, overexpression of PSME1‐2/PA28αβ and its consequent increased association with the 20S proteasome correlated with elevated proteasomal processing activity and accelerated clearance of aggregation‐prone UPS reporters, as well as mutant αB‐crystallin aggregates [41, 149]. Moreover, in a mouse model of inherited retinal degeneration, driven by mutant rhodopsin, overexpression of PSME1/PA28α alone enhanced proteolytic processing and improved photoreceptor function [150]. It is also worth mentioning that, beyond selectively enhancing degradation patterns, overexpression of PSME1/PA28α in mice also resulted in reduced age‐related cognitive decline and preserved explorative behavior [151]. However, PSME1/PA28α overexpression in HD mice did not enhance the degradation of mutant huntingtin, despite increased proteasomal cleavage of a polyglutamine peptide observed *in vitro* [152].

The third member of the family, PSME3/PA28γ, assembles into a homo‐heptameric ring that associates with the 20S core particle [36], leading to allosteric activation of the proteasome's trypsin‐like activity [40]. Unlike PSME1/PA28α and PSME2/PA28β, expression of PSME3/PA28γ is predominantly localized in the nucleus, where it preferentially stimulates degradation of intrinsically disordered or unstructured substrates [153]. During bacterial infections, PSME3/PA28γ binding to the 20S core proteasome is enhanced and accompanied by changes in peptide cleavage patterns that contribute to antimicrobial activity [154]. In *C. elegans*, cold exposure also induces transcriptional upregulation of PSME3/PA28γ, resulting in enhanced trypsin‐like activity and selective clearance of aggregation‐prone substrates, thereby suppressing pathological protein aggregation [155]. Improvements in UPS activity were also observed following lentiviral overexpression of PSME3/PA28γ in a HD mouse model, which resulted in a decrease in ubiquitin‐positive aggregates and improved motor coordination [156]. However, the role of PSME3/PA28γ‐capped proteasomes in HD has been controversial as it had been earlier proposed that they may contribute to the pathogenesis. It was speculated that the reduction in chymotrypsin‐ and caspase‐like activities of PSME3/PA28γ‐capped proteasomes could result in diminished cleavage of expanded polyglutamine repeats within mutant huntingtin, leading to inefficient processing of the pathogenic protein and subsequent clogging of proteasomes [157]. However, reducing the levels of PSME3/PA28γ in a HD mouse model did not ameliorate the disease phenotype, undermining the rationale for PSME3/PA28γ therapeutic inhibition [158]. It should be noted though that later studies were unable to provide evidence for global impairment of the UPS in mouse models of HD [159, 160] and SCA type 7 [161], despite a transient reduction in ubiquitin‐dependent proteasomal degradation observed upon acute expression of mutant huntingtin in cellular and mouse models for HD [162, 163]. Thus, while the presence of aggregation‐prone mutant huntingtin does not appear to be without consequence for UPS function, these studies argue against the role of proteasome dysfunction in polyglutamine repeat disorders.

PSME4/PA200 is another regulator that can associate with the 20S core [44]. Unlike other proteasome caps, this unusually large protein binds as a monomer, and its role in proteasome activation is still not clear. Like PSME3/PA28γ, it localizes predominantly to the nucleus and modifies the proteolytic profile, leading to a relative increase in caspase‐like activity and resulting in enhanced protein degradation *in vitro* [164, 165]. PSME4/PA200 is also upregulated in various cancers, where it reprograms proteasome activity by favoring caspase‐like over trypsin‐like cleavage. This altered cleavage specificity changes the repertoire of peptides generated by the proteasome, thereby contributing to immune evasion of malignant cells [166].

While several peptides and compounds enhance proteasomal degradation by promoting opening of the 20S gate, additional small molecules have been identified that activate proteasome function through distinct, gate‐independent mechanisms. Among them, sulforaphane, one of the earliest small molecules reported to stimulate proteasome activity, induces the expression of catalytic proteasome subunits and increases cellular resistance to oxidative stress [167]. Another example is betulinic acid, which selectively enhances the chymotrypsin‐like activity of the 20S proteasome *in vitro* [168], without affecting protein degradation in cellular systems [99]. Finally, ketamine, which is an antagonist of N‐methyl‐D‐aspartate receptors, stimulates proteasomal activity within hours after administration [169]. Since ketamine and its related compounds already have regulatory approval for the treatment of depressive disorders, they represent particularly attractive candidates for therapeutic repurposing. It, however, remains unclear whether the ketamine‐induced increase in catalytic activity translates into enhanced degradation of specific protein substrates *in vivo* and, if so, what those substrates are [169].

## Stimulation beyond the proteasome: Extraction and ubiquitin shuttling factors

Beyond the proteasome itself, enhanced UPS activity can also be driven by upstream regulatory factors. One such key regulator is the ubiquitin‐selective segregase p97/VCP, an AAA‐ATPase whose catalytic domains resemble those of the 19S regulatory particle. P97/VCP assembles into a hexameric, ring‐shaped complex with a central pore, composed of an N‐terminal domain and two ATPase domains, D1 and D2 [170]. The p97/VCP complex functions in concert with various adaptor proteins, most notably the ubiquitin‐binding UFD1/NPL4 heterodimer, which is essential for processing ubiquitinated proteins. Although p97/VCP can transiently interact with the 20S core particle, promoting gate opening and facilitating substrate engagement [171, 172, 173], it primarily operates as an independent upstream unfoldase, which extracts and remodels ubiquitinated proteins before their delivery to the proteasome [174].

In addition to p97/VCP's capacity to segregate and extract ubiquitinated proteins from multiprotein assemblies or membranes, its ability to unfold substrates has a broader functional significance. By unfolding client proteins, p97/VCP generates the unstructured initiation sites required by the 19S regulatory particle to begin translocation of captured substrates into the proteasome [175]. A key mechanistic distinction between p97/VCP and the AAA‐ATPases of the proteasome is that p97/VCP can initiate unfolding from a proximal ubiquitin [176], rather than requiring a pre‐existing unstructured initiation region. This allows p97/VCP to act on tightly folded substrates that the proteasome alone cannot readily process. To promote the translocation of substrates through the 20S central pore, p97/VCP starts by unfolding the initiator ubiquitin molecule followed by the substrate and culminating in the release of the unfolded polyubiquitinated substrate, which can then be shuttled to the proteasome for degradation [177]. Substrates that already contain sufficiently loose or intrinsically disordered regions can be degraded independently of p97/VCP [178] and may be less reliant on polyubiquitination [179]. Importantly, in addition to its critical role in proteasomal degradation, p97/VCP is also involved in other protective mechanisms against aggregation‐prone proteins, most notably autophagy, where it participates in maintaining proteostasis under cellular stress conditions [180, 181].

As p97/VCP drives the extraction and unfolding of ubiquitinated proteins, enhancing its activity could, in principle, accelerate the clearance of misfolded substrates. Moreover, mutant huntingtin with expanded polyglutamine repeats has been shown to impair UPS function by interfering with the functionality of the p97/VCP complex [182], providing an additional rationale for therapeutic strategies aimed at restoring or boosting p97/VCP function. Supporting this idea, p97/VCP overexpression has yielded beneficial effects in multiple disease models expressing aggregation‐prone proteins. In a zebrafish model of frontotemporal dementia (FTD), elevating p97/VCP levels reduced mutant tau accumulation [183]. Similarly, in a mouse model of amyotrophic lateral sclerosis (ALS) expressing mutant SOD1, p97/VCP overexpression extended the lifespan [184]. At the cellular level, mutant SOD1 disrupts and delays the degradation of several proteins, including p97/VCP itself, resulting in dysregulated p97/VCP homeostasis [185]. Restoring p97/VCP levels through ectopic overexpression recovered proteostasis by normalizing degradation dynamics and reducing the buildup of insoluble SOD1 [185].

The p97/VCP complex contains multiple druggable sites, and several small‐molecule modulators have been developed that modulate its ATPase activity. For example, the compounds NW1030 [186] and SMER28 [187] stimulate p97/VCP by binding at the interface between the N‐terminal region and the D1 ATPase domain. SMER28 was initially identified as an autophagy activator [188], but was later shown to directly interact with p97/VCP [187], a finding that likely explains its biological activity given p97/VCP's known role in promoting autophagy [180, 181]. Activation of p97/VCP by SMER28 promotes the clearance of aggregation‐prone proteins, including mutant huntingtin and α‐synuclein [187]. Another compound, UP109, also targets p97/VCP's ATPase activity but binds instead to the D2 ATPase domain [189]. Administration of UP109 reduces the levels of intranuclear TDP‐43 aggregates, highlighting its potential therapeutic relevance [189]. Finally, the allosteric activator VAA1 binds to a pocket near the C terminus of p97/VCP, providing yet another mechanism for stimulating ATPase activity [190].

Mutations of p97/VCP are associated with several neurological and non‐neurological diseases characterized by the accumulation of misfolded proteins. The most prominent examples include ALS [170] and multisystem proteinopathy (MSP), which is a condition characterized by frontotemporal dementia combined with muscle and bone pathology [177]. Pathogenic variants of p97/VCP typically cluster within hotspot regions of the N‐terminal and D1‐ATPase domains [191, 192], where they can interfere with p97/VCP physiological functions by impairing autophagy [180], disrupting interactions with essential cofactors [193, 194], or increasing its ATPase activity [195, 196]. At first glance, it may seem paradoxical that elevated ATPase activity contributes to pathology [170, 177], while pharmacological stimulation has been proposed as therapeutically beneficial [183, 184, 185, 189]. A possible explanation may lie in the dual nature of protein unfolding: Although p97/VCP‐mediated unfolding is required for efficient proteasomal degradation of many substrates, excessive unfolding can convert otherwise stable proteins into aggregation‐prone intermediates, thereby worsening proteotoxic stress rather than alleviating it. This suggests that stimulation of p97/VCP is likely to be beneficial only when the rate of substrate unfolding does not exceed the degradative capacity of the UPS. Consequently, caution is warranted when attempting to compensate for reduced UPS activity by further boosting p97/VCP‐driven substrate unfolding.

Modulating the activity of ubiquitin shuttle factors that deliver ubiquitinated proteins to the proteasome provides another possibility to promote degradation [197]. Rad23, initially characterized as a protein important for nucleotide excision repair, was later found to mediate ubiquitin‐dependent proteasomal degradation [198]. Its contribution to proteostasis is multifaceted: Rad23 can promote degradation by simultaneously engaging with ubiquitinated substrates through its UBA domain and with the proteasome via its UBL domain [199]. Conversely, Rad23 can also inhibit degradation, either by sequestering ubiquitinated substrates [200] or by stabilizing specific proteins as in nucleotide excision repair [201]. The physiological relevance of Rad23 modulation has been demonstrated in multiple disease models. Deletion of the rad‐23 orthologue in *C. elegans* expressing mutant SOD1 or TDP‐43 suppressed motility defects and proteotoxicity, an effect attributed to enhanced clearance of the mutant proteins [202]. A comparable effect was observed in human and mouse cell lines upon Rad23 depletion [202]. In an *in vivo* system, a similar protective outcome was attributed to deletion of the Rad23A orthologue that ameliorated TDP‐43 pathology in mice [203]. Rad23 also interacts with ataxin‐3, and its depletion reduced toxicity associated with mutant ataxin‐3 in a *Drosophila* model [204]. Although the molecular mechanism is poorly defined and it is presently unknown whether a shared mechanism is responsible for the effects on SOD1, TDP43 and ataxin‐3, these studies hint that modulation of ubiquitin shuttle factors may be a therapeutic interest.

## Stimulation beyond the proteasome: Modulating substrate accessibility

As the process of proteasomal degradation requires protein unfolding to allow the translocation of the substrate into the proteolytic chamber, processing hard‐to‐unfold substrates, such as protein aggregates, presents a major challenge. Indeed, impairment of the UPS observed during acute expression of aggregation‐prone proteins [162, 163] has been proposed to arise from proteasomes getting stuck in their attempts to degrade these refractory substrates [205]. One potential mechanism to alleviate this deadlock is stimulating the disaggregation of such proteins, which would convert them into amenable proteasome substrates. Here, p97/VCP plays a key role, as it has been shown to cooperate with Hsp70 in promoting the disassembly and subsequent proteasomal clearance of Tau aggregates [206]. However, p97/VCP action potentially comes with other consequences, as it can enhance the propagation of aggregates through the concomitant generation of seeding‐competent Tau species [206, 207].

The proteasome itself possesses both chaperone activity [208] and disaggregation capacity [209, 210]. Even though the proteasome may not be able to fully unfold protein aggregates into conformations compatible with proteasomal degradation, a recent study suggests that it nevertheless can generate fragments of cytosolic aggregates and facilitate their clearance by autophagy, in conjunction with the DNAJB6‐HSP70‐HSP110 chaperone system [209]. In the nuclear compartment, instead, the HSP70‐HSP110 machinery cooperates with ubiquilin‐2 (UBQLN2), which directly binds to HSP70 to deliver disaggregated proteins to the proteasome for ubiquitin‐dependent degradation [211].

Beyond the intrinsic resistance of protein aggregates to unfolding, their subcellular localization can present another barrier to efficient proteasomal targeting and clearance. Prime examples are aggregation‐prone proteins that reside in the ER, as they must be first translocated into the cytosol before they become accessible for degradation. For instance, the rhomboid protease RHBDL4 appears to play a general role in promoting the degradation of ER‐resident aggregation‐prone proteins by cleaving them in the lumen of the ER, thereby promoting their p97/VCP‐dependent extraction into the cytosol, where they become accessible to the proteasome [212]. When RHBDL4 is overexpressed, this clearance pathway is further enhanced, accelerating the degradation of misfolded ER luminal proteins and preventing their aggregation [212].

Several aggregation‐prone proteins implicated in neurodegenerative diseases have been found to mislocalize to mitochondria [213, 214, 215]. This is likely a direct consequence of their hydrophobic properties, which can promote interactions with lipid membranes [216, 217, 218], but may also stem from the similarities of their amphipathic helices to mitochondrial localization signals [219]. Interestingly, genetic or chemical inhibition of the translation factor eIF5A, a regulator of mitochondrial homeostasis [220], limits the localization and sequestration of these proteins at mitochondria, promoting their proteasomal degradation [221]. These findings suggest that mitochondria may act as a protective niche for aggregation‐prone proteins, where they are relatively shielded from the ubiquitination machinery and, consequently, inefficiently targeted for proteasomal clearance [221].

## Targeted protein degradation

One potential limitation of globally stimulating proteasome activity is the lack of substrate specificity, since an ideal strategy would preferentially direct UPS activity toward disease‐associated proteins only. Rather than broadly enhancing UPS function, an alternative and increasingly attractive approach is based on promoting the selective delivery of proteins to the proteasome by modulating their ubiquitination. In this context, there is a growing interest in using small molecules in targeted protein degradation strategies, including proteolysis‐targeting chimeras (PROTACs) [222] and molecular glues [223]. These molecules enable selective substrate degradation by inducing or stabilizing interactions between ubiquitin ligases, such as Cereblon (CRBN) [224, 225] and Von Hippel–Lindau (VHL) [226], and proteins of interest, thereby enhancing their ubiquitination and subsequent clearance [227, 228]. CRBN and VHL are components of ubiquitin ligase complexes that primarily assemble canonical K48‐linked ubiquitin chains on substrates [229, 230].

Interestingly, the K29 linkage–specific ubiquitin ligase TRIP12 cooperates with PROTAC‐recruited E3 ligases, such as VHL, to generate branched K29/K48‐linked chains [230]. This observation led to the suggestion that branched ubiquitin chains may compensate for suboptimal ubiquitination of neo‐substrates by PROTAC‐recruited ubiquitin ligases. It has been shown that preventing deubiquitination by proteasome‐associated DUBs preferentially enhances degradation of aggregation‐prone proteins, suggesting that robust ubiquitination may be particularly important for these substrates, similar to the neo‐substrates [136, 142]. This raises the possibility that PROTAC‐induced formation of branched chains may be particularly advantageous for targeting this type of aggregation‐prone substrates. Notably, also K11/K48‐branched ubiquitin chains represent an alternative degradation signal associated with enhanced protein turnover [231, 232] and may therefore offer further opportunities for optimizing PROTAC design.

Targeting proteins for degradation with conventional PROTACs and molecular glues depends on the availability of ubiquitin and the proper functioning of the ubiquitination machinery. Although ubiquitin is abundantly expressed, proteotoxic stress conditions lead to a marked accumulation of ubiquitinated proteins, which results in depletion of the pool of free, uncommitted ubiquitin [233]. This reduction selectively impairs the clearance of aggregation‐prone proteins [234]. Consequently, strategies that bypass the requirement for ubiquitination may offer a distinct benefit for pharmacologically induced protein degradation. A recently developed midnolin‐based targeted protein degradation strategy substantially advances the feasibility of this approach [235]. Midnolin directs nuclear proteins to the proteasome for ubiquitin‐independent degradation by simultaneously binding the substrates and the proteasome, thereby promoting proteasomal engagement through its UBL domain [236, 237]. As a proof of principle, midnolin‐based targeting chimeras (MbTACs) can increase ubiquitin‐independent proteasomal degradation of c‐Myc or androgen receptor [235]. MbTACs may represent an attractive strategy for targeting misfolded proteins implicated in neurodegenerative disorders as well; however, it remains to be seen whether this approach can be generalized for aggregation‐prone proteins.

Another strategy aimed at directly targeting substrates to the proteasome takes advantage of a ligand that binds the PSMD2/Rpn1 subunit and promotes ubiquitin‐independent degradation of BRD4, a chromatin regulator and oncogenic driver [238]. This bifunctional construct positions the BRD4 substrate in close proximity to the AAA‐ATPase subunits of the 19S proteasome cap, enabling subsequent proteasomal engagement and degradation [238]. A related approach employs a ligand that binds the proteasome‐associated DUB USP14 to target oncogenic proteins for ubiquitin‐independent degradation [239]. Although these strategies bypass the need for ubiquitination, they still rely on fully active proteasomes. Accordingly, combining targeted protein degradation approaches with general proteasome activators may produce synergistic effects.

## Concluding remarks and outlook

In contrast to the successful clinical introduction of proteasome inhibitors as first‐line therapies for hematological malignancies [240], small molecules designed to enhance UPS activity have not yet progressed from bench to bedside. Although conceptually appealing, several factors have hindered the development of proteasome stimulators. Most importantly, creating drugs that activate proteasome function is inherently more challenging than developing inhibitors, as small molecules typically exert their effects by blocking catalytic activities rather than enhancing them. Furthermore, in neurodegenerative diseases and other human proteinopathies, effective treatment would require chronic administration at an early stage to counteract the age‐related accumulation of misfolded proteins. While prophylactic treatment may be feasible for familial variants, sporadic cases present an additional obstacle as the UPS must contend with pre‐existing protein aggregates, which are intrinsically poor proteasome substrates due to their aggregation and resistance to unfolding.

Sustained activation of the 26S proteasome may also have unintended consequences. Key regulatory proteins, such as p53 [241] and components of the NF‐κB pathway [242], which are normally stabilized in response to cellular stresses, may be hindered by persistently elevated proteasome activation and potentially compromise these regulatory processes. In addition, excessive turnover of cell‐cycle regulators may disrupt cell division checkpoints [243]. Together, these effects may impair the cell's ability to mobilize protective stress responses. However, enhanced proteasome activity does not necessarily translate into accelerated degradation of these substrates, as degradation kinetics are often primarily dictated by ubiquitination rather than proteasome availability. Indeed, *in vivo* studies examining proteasome activation indicate that the potential benefits, such as improved clearance of aggregation‐prone proteins [65, 66, 67, 131] or reversing UPS impairment [123, 203], most likely outweigh potential adverse effects.

The turnover of long‐lived proteins, protein aggregates, and entire organelles is primarily mediated by autophagy. Although the reciprocal crosstalk is rather well‐characterized when either system is inhibited [244], the consequences of proteasome stimulation on autophagy are far less explored. Enhancing proteasomal degradation of aggregation‐prone proteins could, in principle, reduce the dependence on autophagy for aggregate clearance and thereby increase autophagic capacity. However, this relationship is not straightforward due to the tight functional interconnection between these two systems. Indeed, it has been suggested that proteasome stimulation via a compound that induces a constitutively open 20S proteasome blocks the final step of autophagosome–lysosome fusion due to increased turnover of SNARE proteins [245].

Several studies suggest that under physiological conditions, as little as 20% of total proteasome capacity is sufficient to prevent the accumulation of ubiquitinated proteins [246, 247, 248]. The large surplus of proteasome activity under physiological conditions may reflect the difficulties cells would face if it would instead forced to rapidly upregulate proteasome levels during acute proteotoxic stress. This stands in sharp contrast to ubiquitin, whose cellular concentration can be quickly increased and immediately used in response to proteotoxic stress. Indeed, data indicate that during the early stages of acute stress, it is ubiquitin availability, rather than proteasome abundance, that limits UPS function [234]. Hence, it may be beneficial to target at the same time the availability of ubiquitin while enhancing proteasome activity. Yet, both *in vitro* and *in vivo* studies demonstrate that increasing or modulating proteasome activity (Table 1) as well as substrate delivery can accelerate degradation, including that of aggregation‐prone proteins implicated in neurodegenerative diseases, suggesting that stimulation of proteasomal degradation may hold therapeutic potential.

**Table 1:** List of genes and compounds that activate proteasomal degradation.

Modification type	Activator	Strategy	Effect on protein degradation or physiology	References
Changes in 20S	PSMB5/β5	Overexpression	Stress resistance	[60]
	PSMB5/β5	Overexpression	Age‐related decline restored	[61]
	PSMB5/β5	Overexpression	Stress resistance	[62]
	PSMB5/β5	Overexpression	Age‐related decline decreased	[63]
	PSMB5/β5	Overexpression	Life span increased	[65]
	PSMB5/β5	Overexpression	Age‐related decline	[64]
	PSMB5/β5	Overexpression	Life span increased	[66]
	Nt deletion PSMA4/α3	Gate open mutant	Aggregation‐prone proteins	[79]
	Nt deletion PSMA4/α3	Gate open mutant	Stress resistance and life span increased	[80]
	Nt deletion PSMA4/α3	Gate open mutant	Misfolded and intrinsically disordered proteins	[81]
	POMP	Overexpression	Stress resistance increased	[70]
	POMP	Overexpression	Stress resistance increased	[69]
	ASC‐JM1	NRF1 activator	Mutant androgen receptor	[75]
	RUN‐47	NRF1 activator	α‐synuclein	[76]
Changes in 20S (peptide)	Hydrophobic peptides	Gate open		[85]
	HbYX‐like dipeptide	Gate open	Tau	[87]
	Ct PSME4/PA200‐based peptide	Gate open		[89]
	Rpt5‐based peptide	Gate open	α‐synuclein, tau	[88]
	Cyclic peptides	Gate open	Intrinsically disordered proteins	[92]
	PSME/PA28‐based peptides	Gate open		[249]
	PAP1 peptide	Gate open	Oxidized proteins, SOD1	[93]
Changes in 20S (compounds)	MK‐866, AM‐404	Gate open	α‐synuclein	[99]
	Syrosingopine	Gate open	α‐synuclein	[98]
	Chlorpromazine	Gate open	α‐synuclein, tau	[95]
	Fluspirilene	Gate open	Amyloid β‐42, α‐synuclein	[97]
	DBTO	Gate open	Amyloid β‐42, life span increased	[100]
	Oleuropein	Gate open	Life span increased	[94]
	TCH‐165	Gate open	α‐synuclein, tau	[96]
Changes in 19S	PSMD11/Rpn6	Overexpression		[104]
	PSMD11/Rpn6	Overexpression	Stress resistance and longevity, polyQ proteins	[103]
	PSMD5/S5b	Depletion	UPS reporter, tau	[105]
	PSMD5/S5b	Depletion	Aggregation‐prone proteins	[106]
	AIRAP/ZFAND2A	Stress induction		[111]
	ZFAND5	Overexpression	Overall proteins	[55, 56]
	Forskolin, PSMC5/Rpt6	cAMP‐PKA induction		[121]
	Db‐cAMP, Forskolin, Rolipram	cAMP‐PKA induction	Ubiquitinated proteins	[122]
	Sildenafil	cGMP‐PKG induction	UPS reporter, CryAB^R120G^	[127]
	Rolipram	cAMP‐PKA induction	Aggregation‐prone proteins	[120]
	Rolipram	cAMP‐PKA induction	Tau	[123]
	Glucagon, epinephrine, Forskolin	cAMP‐PKA induction	Short‐lived proteins	[125]
	Tadalafil, Sildenafil, BAY41‐2272	cGMP‐PKG induction	Cytosolic proteins, tau	[126]
Other regulatory caps	PSME1‐2/PA28αβ	Overexpression	UPS reporter, oxidized proteins	[41]
	PSME1‐2/PA28αβ	Overexpression	UPS reporter, CryAB^R120G^	[149]
	PSME1‐2/PA28αβ	Overexpression	Improved photoreceptor survival	[150]
	PSME3/PA28γ	Overexpression		[40]
	PSME3/PA28γ	Cold temperature	PolyQ proteins, life span increased	[155]
	PSME4/PA200	Overexpression	Tau	[164]
Proteasome‐associated DUBs	USP14	Depletion	Tau, ataxin‐2	[136]
	IU1	Inhibition	Tau, ataxin‐3	[136]
	IU47	Inhibition	Tau	[139]
	IU1	Inhibition	Prevented neuronal death	[138]
	UBL domain USP14	Overexpression	Ubiquitinated proteins	[135]
	UCHL5	Depletion	UPS reporter	[142]
Other small molecule inhibitors	PD163916 (p38 depletion)	Inhibition	α‐synuclein	[128]
	Sulforaphane	Inhibition	Oxidative stress	[167]
	Betulinic acid	Inhibition		[168]
	Ketamine	Inhibition		[169]
	Rapamycin, Torin1	Inhibition	Short ‐ and long‐lived proteins	[132]
	IGF‐1	Supplementation	Oxidized proteins	[131]

Many challenges undoubtedly remain, but with the collective efforts of the numerous laboratories investigating the intricate relationship between the UPS and human proteinopathies and exploring strategies to modulate and harness this system, the hope persists that the unique capabilities of this powerful machinery can ultimately be leveraged in a therapeutic context.

## Conflict of interest

The authors declare no conflict of interests.

## Author contributions

MEG and NPD wrote the review draft. EB generated the figures. All authors edited and approved the final manuscript.
